# The Future of Enhanced Recovery After Surgery in General Surgery: Integrating Artificial Intelligence, Personalized Care, and Technological Advances

**DOI:** 10.7759/cureus.91528

**Published:** 2025-09-03

**Authors:** Mohamed Abosheisha, Elmoatazbellah Nasr, Mohamed Abdellatif, Ahmed Swealem, Ahmed Ammar, Md Abdus Samad Hasan, Momen Abdelglil, Rezuana Tamanna, Mohamed Ismaiel

**Affiliations:** 1 General Surgery, Wirral University Teaching Hospital NHS Foundation Trust, Wirral, GBR; 2 General Surgery, Calderdale and Huddersfield NHS Foundation Trust, Huddersfield, GBR; 3 General Surgery, University Hospitals of Leicester NHS Trust, Leicester, GBR; 4 Orthopedics, North Bristol Foundation Trust, Bristol, GBR; 5 General Surgery, Sunderland Royal Hospital, Sunderland, GBR; 6 Medicine, Bangkok Hospital, Bangkok, THA; 7 Pediatric Surgery, Mansoura University Children Hospital, Mansoura, EGY; 8 General Surgery, Watford General Hospital, Watford, GBR; 9 Surgery, University Hospital Limerick, Limerick, IRL

**Keywords:** artificial intelligence, enhanced recovery after surgery, eras, general surgery, implementation science, perioperative care, precision medicine

## Abstract

Enhanced Recovery After Surgery (ERAS) protocols have changed and improved surgical care and practice, transforming classic approaches toward more evidence-based practices. ERAS protocols demonstrate consistent benefits, including reduced hospital length of stay, decreased postoperative complications, and significant cost savings. Advances include the development and integration of AI and digital health technologies that promise to personalize and optimize recovery pathways. However, the global application of ERAS may face great challenges, starting with resource limitations, resistance to change, and variable compliance rates. This review discusses and highlights the application of ERAS, identifies barriers to implementation, and proposes evidence-based recommendations for optimizing ERAS adoption and sustainability in modern surgical practice.

## Introduction and background

Over the past two decades, Enhanced Recovery After Surgery (ERAS) has transformed surgical practice [1]. The fundamental principle underlying ERAS is the reduction of surgical stress response through multiple techniques that adjust physiological function and accelerate recovery after surgery [2,3].

ERAS protocols are adjusted and designed for three distinct phases of care: preoperative preparation, intraoperative management, and postoperative optimization. Each phase incorporates evidence-based interventions designed to minimize physiological disruption, preserve organ function, and facilitate rapid return to baseline activity levels [4,5].

Previous systematic analyses have highlighted that the ideal usage of ERAS protocols led to shortened hospital stays, decreased healthcare costs, and improved patient satisfaction [6].

Then, the ERAS society has developed protocols that focus on measures that help in early recovery. Adherence to ERAS protocols reduces postoperative complications by up to 50% and shortens the length of hospital stay and the need for admission by two to three days across a range of general surgical procedures [7].

While ERAS consistently improves perioperative recovery and efficiency, its global expansion remains constrained by uneven resources, cultural resistance, and inconsistent compliance. Accordingly, this review pivots from detailing multiple outcome measures toward examining adoption dynamics. We highlight key trends such as the integration of digital health, artificial intelligence (AI), and tele-prehabilitation that have the potential to support broader implementation. At the same time, we analyze structural and contextual barriers that limit ERAS feasibility at national and global levels, distilling facilitators (e.g., leadership support, tailored adaptation, and training) that can accelerate sustainable implementation.

## Review

Overview of recent advances in ERAS protocols 

Over the past five years, ERAS has reinforced its role in perioperative care by combining evidence-based, multidisciplinary strategies with patient-centered approaches, focusing on optimized interventions, education, multimodal pain control, early discharge, and continuous quality improvement [8,9]. 

Recent advances include the integration of digital health technologies, personalized care strategies, and minimally invasive surgical techniques, all of which help in the enhanced performance and early recovery [8]. Despite these successes, challenges remain in implementation, particularly regarding protocol adherence, resource variability, and the need for further standardization across specialties [10]. 

The future of ERAS is poised to leverage AI, remote monitoring, and tailored and adjusted machine learning (ML) and surgical interventions to further enhance recovery and patient outcomes [8]. The last five years have solidified ERAS as a cornerstone of modern perioperative care, with robust evidence supporting its effectiveness in reducing complications, shortening hospital stays, and improving patient satisfaction across multiple surgical specialties. The multidisciplinary, protocol-driven approach ensures that best practices are consistently applied, while continuous auditing and feedback drive ongoing improvements [10,11].

Advances in technology, such as AI and robot-assisted surgery, are beginning to reshape ERAS pathways, offering opportunities for more personalized, data-driven care. These innovations promise to enhance recovery further, reduce complications, and optimize resource utilization, but they require careful integration into existing protocols and workflows. Ongoing research is needed to evaluate the impact of these technologies and to address barriers to widespread adoption, particularly in resource-limited settings [8].

Integration of AI with ERAS

AI technologies are being leveraged to optimize ERAS protocols by enabling patient risk stratification, personalized care plans, and outcome plan prediction. By analyzing large datasets, AI can predict individual patient risks and tailor interventions, improving adherence and resource allocation [12,13].

Perioperative AI Integration

AI supports preoperative planning by helping in risk assessment and planning, intraoperative guidance via real-time navigation and robotic assistance, and postoperative management with predictive analytics and remote monitoring. These applications enhance surgical precision, reduce complications, and facilitate early detection of adverse events [14-16].

Robotic systems enable complex procedures to be performed minimally invasively, which is a core ERAS recommendation. This approach reduces trauma, the need for long admissions, and postoperative pain, while improving patient satisfaction and potentially lowering complication rates [17,18].

The integration of robotic surgical platforms with ERAS protocols has demonstrated better results compared to classic approaches. Studies show that robotic surgery implementation within ERAS frameworks requires approximately 31 patients to achieve higher compliance rates, with robotic platforms showing reduced postoperative length of stay outcomes compared to laparoscopic approaches (odds ratio = 5.029, 95% CI = 1.321-19.142; *P* = 0.018) [19].

Minimally invasive colorectal surgery combined with ERAS protocols achieved successful protocol completion in most patients (64.7%), leading to faster recovery times (90.2 ± 98.8 vs. 95.9 ± 33.4 hours; *P* = 0.003). This shows how modern surgical techniques can strengthen the benefits of ERAS [20].

**Figure 1:**
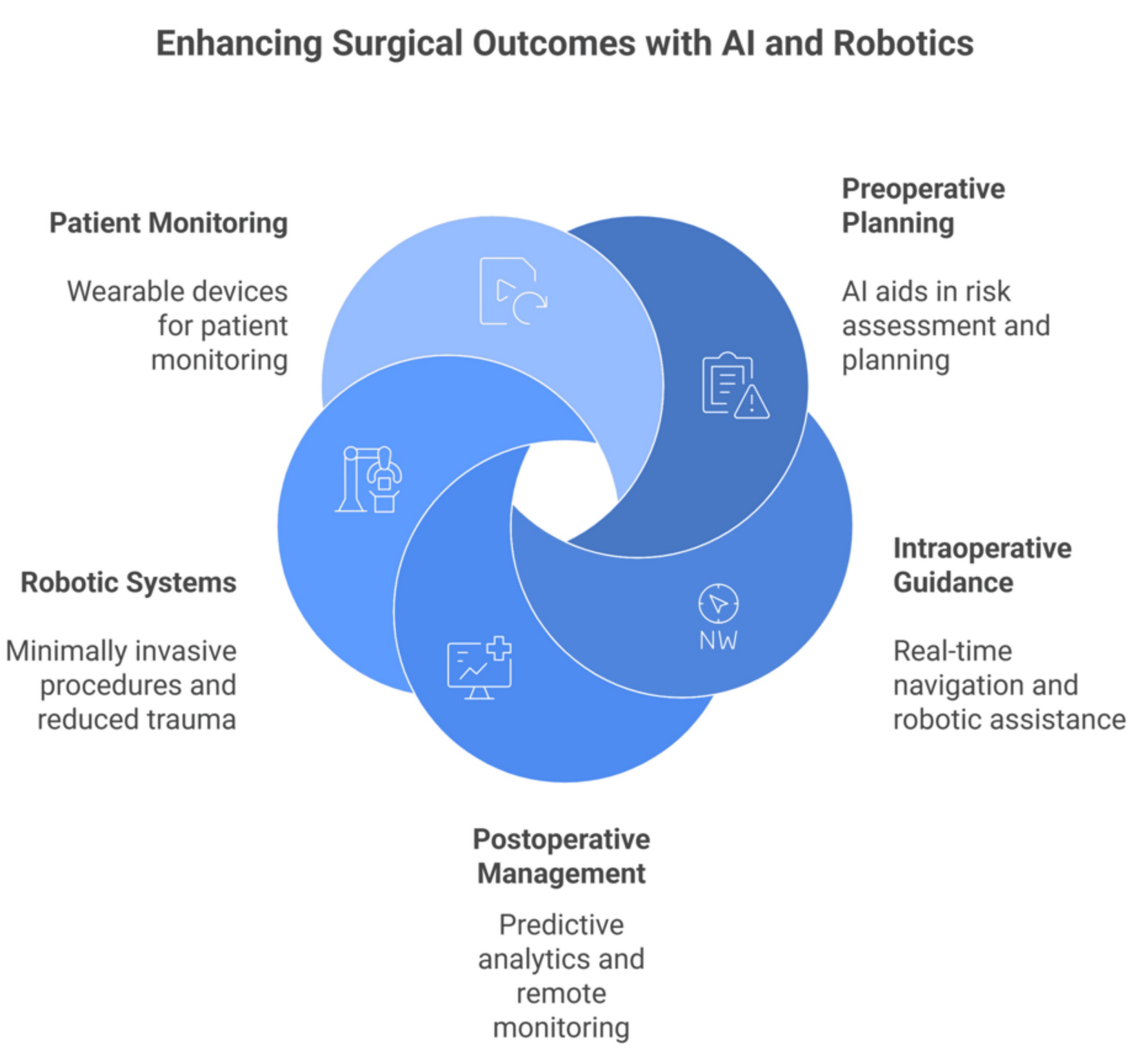
A summary of perioperative artificial intelligence (AI) integration. Image credit: Momen Abdelglil. Sources: [12-21].

Studies across various specialties (urology, gynecology, thoracic, pediatric, and hernia surgery) consistently show that combining robotic surgery with ERAS protocols leads to shorter hospital stays, faster return of bowel function, earlier ambulation, and analgesic needs without other complications [17,21]. High adherence to ERAS protocols in robotic surgery is associated with improved overall survival rates (Figure 1) [21].

Effect on Clinical Outcomes 

Studies demonstrate that AI-driven interventions can reduce overall complications, though the impact on length of stay and readmission rates is variable. ML models have shown high predictive accuracy for postoperative complications and recovery trajectories, but bigger datasets and additional external validation are needed for strong clinical adoption [22-24].

When creating perioperative recommendations, AI-powered large language models (LLMs) and ML tools have shown a high degree of alignment with expert consensus, particularly in orthopedic ERAS protocols. AI systems can support preoperative preparation, intraoperative management, and postoperative care, with ChatGPT outperforming other LLMs in accuracy and clinical relevance [23].

AI enables the development of personalized data recovery plans by integrating data from wearables, electronic health devices and records, and mobile health applications [25,26]. These systems can dynamically adjust protocols based on real-time patient data, optimizing pain management, mobility, and complication prediction, which may reduce recovery time and readmissions [14,27,28].

Studies report that AI integration in ERAS protocols can improve protocol adherence and enhance patient satisfaction [13,28]. In thoracic and colorectal surgery, AI-driven imaging, robotic assistance, and predictive models have improved diagnostic accuracy, operative accuracy, and postoperative recovery [16,27]. However, the quality of evidence varies, with many studies emphasizing the need for external validation and larger datasets [28-30].

Barriers to effective AI integration include data privacy concerns, algorithmic bias, lack of external validation, and the need for physician and nurse training. The literature stresses the importance of transparent communication, ethical views, and collaboration between clinicians and data scientists to ensure safe and effective AI deployment [26,31]. Ethical concerns include transparency, informed consent, and maintaining human oversight in decisions and planning. Addressing these barriers is essential for safe and effective clinical integration [12,30,32].

Digital health platforms for patient monitoring 

As health systems change, digital technological innovations are revolutionizing ERAS implementation, particularly through digital health platforms, minimally invasive surgical technologies, wearable devices, electronic health records, and telemedicine solutions. This transformation is creating new paradigms for patient monitoring, engagement, and recovery pathways [33,34].

Mobile Applications and Patient Engagement

Advances in technology have helped us with several mobile applications designed for ERAS. The iColon application (designed and built by the Operative Unit of General Surgery at the IRCCS Sacro Cuore Don Calabria Hospital, Verona, Italy), tested with 444 colorectal surgery patients, demonstrated significant improvements in ERAS compliance, achieving 74.1% overall adherence to active protocol items. Patients using the application showed a significant reduction in 30-day readmission rates and reported 94% positive satisfaction with the digital platform, with 92.7% indicating improved quality of care perception [35].

The MobERAS gamified mobile application represents another breakthrough in digital health platforms (Universidade Estadual Paulista “Júlio de Mesquita Filho”, Botucatu, Brazil; Pontifícia Universidade Católica de Minas Gerais, Belo Horizonte, Brazil; Universidade Federal de Minas Gerais, Belo Horizonte). Game-like components are incorporated into this telemonitoring system to improve patient motivation and compliance with postoperative care guidelines. The application received positive evaluations from both healthcare professionals and technology specialists for its usability, safety, and potential to increase patient and provider engagement with ERAS programs [36].

Patient-Reported Outcomes Integration

The gathering and evaluation of patient-reported outcomes (PROs) is being transformed by digital platforms. eSyM is a PRO-based cancer symptom management system that is integrated with electronic health records and was created by six different U.S. health systems as part of the National Cancer Institute's (NCI's) Improving the Management of Symptoms during and following Cancer Treatment (IMPACT) Consortium. Using validated PRO-CTCAE (Patient-Reported Outcomes version of the Common Terminology Criteria for Adverse Events) items and wellness questions, eSyM enables patients to report symptoms through secure portals, triggering automated reminders, self-management resources, clinician alerts, and population dashboards. Built using agile, user-centered design, it integrates seamlessly into the Epic EHR to facilitate scalable implementation; however, challenges such as EHR technical constraints, variability among sites, and limited technical staffing were encountered. The program demonstrates the potential of EHR-integrated PRO systems to improve symptom monitoring, streamline clinical workflows, and enhance supportive care for cancer patients [37].

Real-Time Monitoring and Command Centers

**Figure 2:**
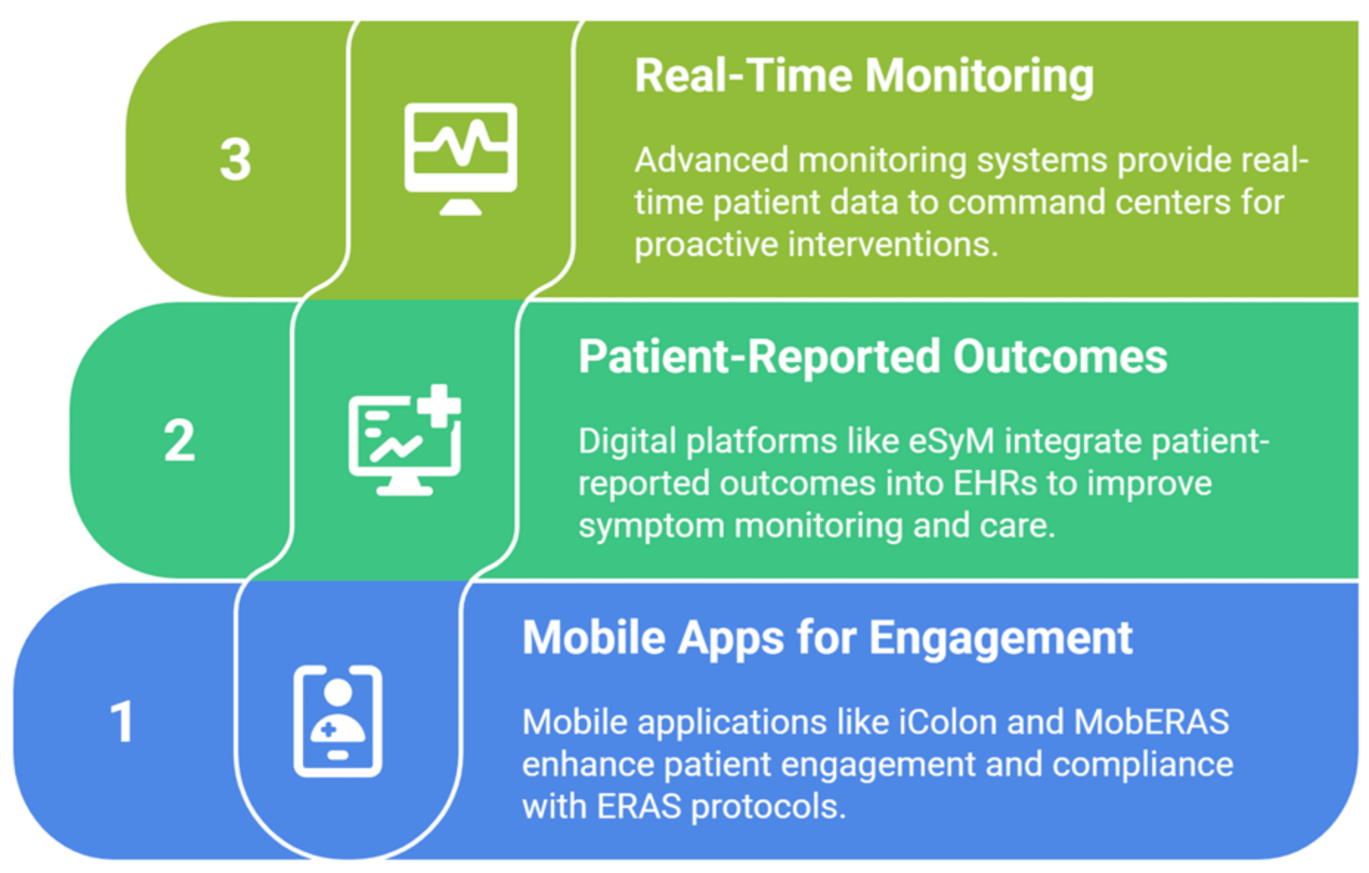
Advancing digital health platforms. Image credit: Momen Abdelglil. Sources:  [32-38]. ERAS, Enhanced Recovery After Surgery; EHR, electronic health record

Advanced digital monitoring platforms enable automatic remote patient surveillance through smart questionnaires and real-time data transmission to hospital command centers. To filter patient-reported data and spot early warning indicators, these systems use complex algorithms. This enables proactive interventions and individualized care modifications. The scalability of these platforms, built on cloud infrastructure, ensures broad accessibility while maintaining data security and patient privacy (Figure 2) [38].

Wearable devices and remote patient monitoring

Wearable devices are transforming perioperative assessment by providing data from patients that are continuous and accurate. These devices enable remote patient monitoring with personalized interventions and proactive healthcare strategies, particularly valuable for pharmacy services and medication adherence tracking [39].

Heart rate variability (HRV) monitoring through wearable devices offers a cost-effective solution for digitalizing perioperative stress monitoring. With the use of this technology, medical professionals can evaluate patients' stress reactions in real time during the perioperative phase and promptly intervene to maximize recovery results [40].

Telemedicine-Enhanced ERAS Programs

Telemedicine is emerging as a valid tool to expand treatment and monitoring outside the hospital setting. Research proved that telemedicine integration with ERAS protocols for minimally invasive mitral valve surgery provides comprehensive patient-centered treatment approaches that address physical, nutritional, and psychological challenges faced before hospitalization and after discharge [41].

The COVID-19 pandemic has accelerated the usage of remote assessment and monitoring devices, creating new opportunities for connected care at a distance. However, successful implementation requires a fundamental shift from episodic care models to continuous connected care frameworks augmented by remote monitoring technologies [42].

Barriers and facilitators to implementation of ERAS

**Figure 3:**
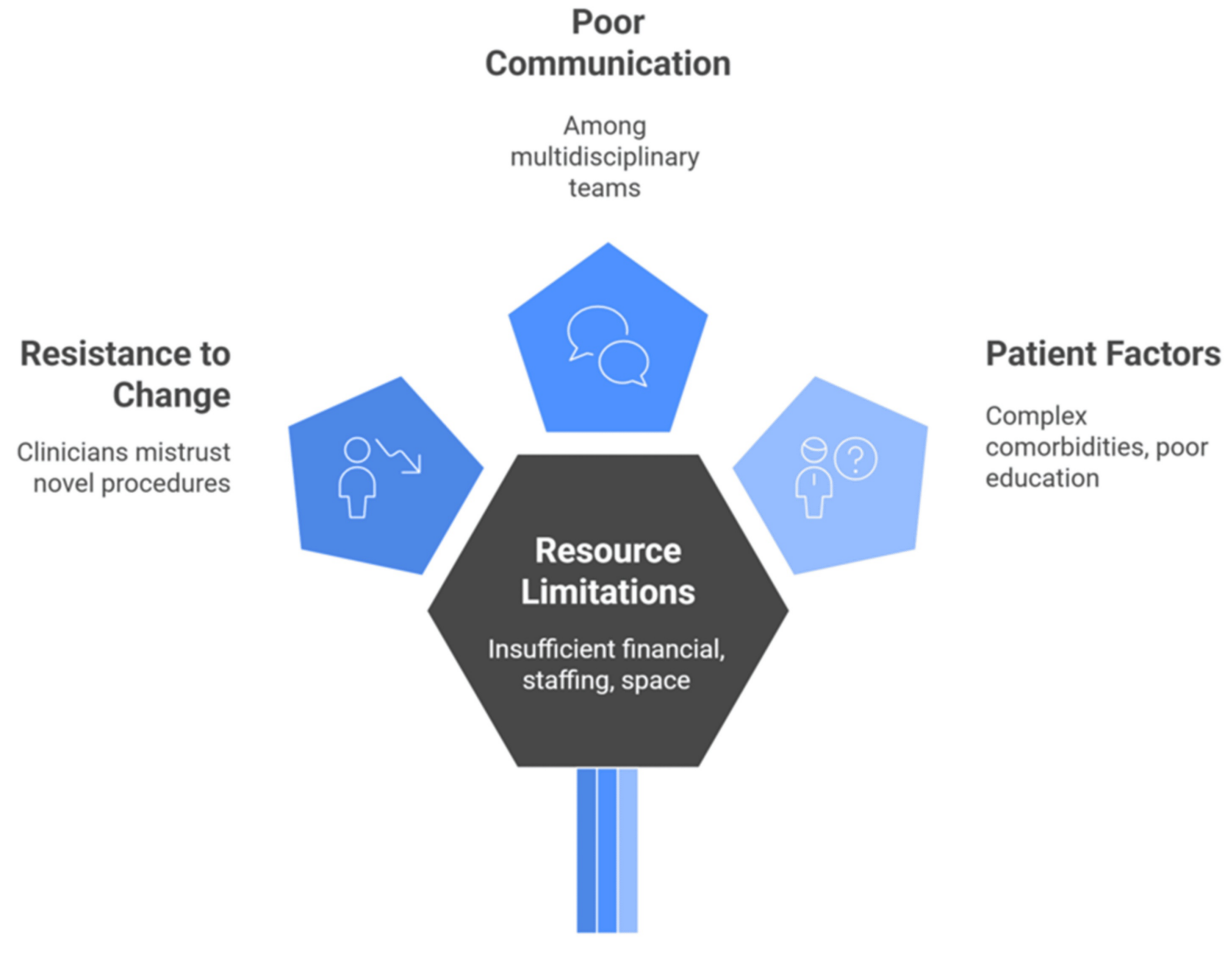
Barriers hindering ERAS globalization. Image credit: Momen Abdelglil. Sources: [42–44]. ERAS, Enhanced Recovery After Surgery

Common barriers to ERAS globalization include resource limitations, such as insufficient financial, staffing, and space resources, along with the absence of dedicated ERAS coordinators, which are particularly challenging in resource-constrained or rural settings. Clinicians and staff frequently exhibit resistance to change, whether active or passive, as a result of ingrained customs or mistrust of novel procedures. Poor communication and coordination among multidisciplinary teams and between departments can further impede adherence. Patient-related factors, including complex comorbidities, socioeconomic challenges, and inadequate patient education, may hinder compliance and affect outcomes. Additionally, system and process issues (Figure 3) [42-44].

Key facilitators for successful ERAS usage between clinicals include strong leadership and team buy-in, with support from hospital leadership, clinical champions, and regular multidisciplinary team meetings driving change. Adapting ERAS protocols to the local context by tailoring them to fit workflows, resources, and patient populations enhances feasibility and adherence. Ongoing education and training for staff, patients, and families about ERAS principles and benefits foster engagement and compliance. Standardization through the use of order sets, checklists, and regular audit and feedback mechanisms ensures consistent practice and continuous improvement. Additionally, achieving early wins and effectively communicating these benefits help build momentum and sustain long-term engagement [43,45,46].

Current gaps and research needs

ERAS protocols have revolutionized perioperative care in general surgery; however, significant research gaps and future directions remain. Adapting protocols for emergency and high-risk surgical populations is a crucial area for future research because the available data is sparse and mostly extrapolated from elective procedures, underscoring the need for randomized controlled trials in these populations. Furthermore, there is a lack of information on long-term outcomes, such as functional recovery, quality of life, and readmission rates, even though the majority of studies focus on short-term advantages like shorter hospital stays and fewer complications. Further research is also needed on digital health integration and strategies to enhance compliance and multidisciplinary collaboration within ERAS programs [47-49].

Future research should focus on personalizing ERAS pathways through digital health devices for patient monitoring and risk assessment, including remote monitoring and patient-specific risk stratification, to optimize recovery and adherence. Sustaining high compliance with ERAS protocols remains a challenge, necessitating studies that identify effective strategies to enhance multidisciplinary teamwork and education and overcome institutional barriers. Additionally, the roles of prehabilitation encompassing preoperative exercise, nutrition, and education, and greater integration of primary care in preoperative optimization show promise but require further evidence to confirm their impact on surgical outcomes [47,50,51].

## Conclusions

ERAS protocols have become a cornerstone of modern surgical practice, offering substantial benefits in terms of patient outcomes and healthcare efficiency. To fully utilize ERAS, the field must overcome a number of significant obstacles. Recurring themes include inconsistent implementation, a lack of high-quality evidence for specific protocol components, and a lack of attention to long-term and patient-centered outcomes. The integration of digital health, AI, and personalized medicine represents a promising frontier but requires careful consideration of ethical, logistical, and training-related barriers.

Global adoption of ERAS is highlighted, with challenges related to resources, infrastructure, and cultural factors. Cross-disciplinary learning and adaptation of protocols from other specialties can help bridge these gaps. Multidisciplinary collaboration, continuous quality improvement, and context-specific research are essential for advancing ERAS implementation and optimizing patient care.
